# Influence of a Biofiller, Polylactide, on the General Characteristics of Epoxy-Based Materials

**DOI:** 10.3390/ma17051069

**Published:** 2024-02-26

**Authors:** Angelika Plota-Pietrzak, Leszek Czechowski, Anna Masek

**Affiliations:** 1 Institute of Polymer and Dye Technology, Faculty of Chemistry, Lodz University of Technology, 90-537 Lodz, Poland; angelika.plota@dokt.p.lodz.pl; 2 Department of Strength of Materials, Lodz University of Technology, 90-537 Lodz, Poland; leszek.czechowski@p.lodz.pl

**Keywords:** epoxy resin, polylactide, biofiller, solar aging

## Abstract

The aim of this work was to obtain epoxy-based composite structures with good mechanical performance, high aging resistance, and an improved degradability profile. For this purpose, powdered polylactide in the amount of 5, 10, 20, 30, and 40 phr was introduced into the epoxy resin, and the composites were fabricated by a simple method, which is similar to that used on an industrial scale in the fabrication of these products. The first analysis concerned the study of the effect of PLA addition to epoxy resin-based composites on their mechanical properties. One-directional tensile tests of samples were performed for three directions (0, 90, and 45 degrees referring to the plate edges). Another aspect of this research was the assessment of the resistance of these composites to long-term exposure to solar radiation and elevated temperature. Based on the obtained results, it was observed that the samples containing 20 or 40 phr of polylactide were characterized by the lowest resistance to the solar aging process. It was therefore concluded that the optimal amount of polylactide in the epoxy resin composite should not be greater than 10 phr to maintain its mechanical behavior and high aging resistance. In the available literature, there are many examples in which scientists have proposed the use of various biofillers (e.g., lignin, starch, rice husk, coconut shell powder) in epoxy composites; however, the impact of polylactide on the general characteristics of the epoxy resin has not been described so far. Therefore, this work perfectly fills the gaps in the literature and may contribute to a more widespread use of additives of natural origin, which may constitute an excellent alternative to commonly used non-renewable compounds.

## 1. Introduction

Plastics are widely used in many industrial applications because of their properties of low weight, high resistance to corrosion and external factors, high stiffness and tensile strength, low production costs, and many others [1]. Epoxy resin belongs to a group of thermosetting polymers that are commonly used in engineering applications due to their high performance (excellent mechanical properties, high thermal and corrosion resistance, etc.) and versatility [2,3,4,5]. It is reported that approximately 323,000 tons of epoxy resin are produced annually, of which 45,000 tons are used in the transportation industry (14%) [6]. The improved characteristics of these products (longer lifetime of vehicles due to greater resistance to corrosion and other factors) contributes to greater savings in raw materials and energy, and as a result, a reduction in the carbon footprint. Nevertheless, the dependence of epoxy resins on nonrenewable resources raises sustainability questions [7]. They are derived from petroleum products that are depleting resources. In addition, their increased production can lead to environmental degradation [8]. Therefore, over recent years, biocomposites containing additives of natural origin have gained a lot of interest in the industry due to EU legal restrictions and growing awareness in the field of environmental protection [9,10].

One example of such an additive to epoxy resin that will partially replace it is biopolymers that are derived from renewable agricultural and biomass feedstock [11,12]. They are gaining importance due to their unique properties, including, above all, high availability, low weight, biocompatibility, biodegradability, and strong barrier capacity [13,14]. These features make them an ideal substitute for petroleum-based products [15]. One of the promising biopolymers is polylactide (PLA), which is a biodegradable and bio-renewable thermoplastic polyester with high mechanical properties. On the other hand, PLA has disadvantages such as high brittleness or low heat resistance. The improvement in PLA properties has been described in several papers [16,17]. Due to the high resistance to heat and corrosion, increased toughness, and low shrinkage of epoxy resin, it has been described as a substance that improves the properties of PLA [18].

Other methods described in the literature to improve the environmental impact of composites based on epoxy resin concerned the introduction of such biofillers into the polymer matrix as nanofibers of cellulose [19], natural short fibers and microfibers from cereal hulls [20], horsetail [21], pine sawdust [22], rice husk [23], acacia catechu particles [24], walnut shell powder [25], eggshell [26], etc. These biofillers improved the performance of the composite material while reducing the costs [27].

In the present study, the effect of a bioadditive in the form of powdered polylactide on the general characteristics of composites based on epoxy resin, was examined. In addition, the manufacturing technique of these materials is simple and at the same time innovative and is similar to that used on an industrial scale in the fabrication of these products. The first analysis concerned the study of the effect of polylactide addition to epoxy resin-based composites on their mechanical properties. Another aspect of this research was the assessment of the resistance of these composites to long-term exposure to solar radiation and elevated temperature. For this purpose, the produced materials were aged in a solar chamber for 800 h at a temperature of 70 °C. The presented approach has not been described in the literature before; therefore, it is definitely a scientific novelty and a good alternative to composites that are a threat to the natural environment.

## 2. Materials and Methods

### 2.1. Materials

An epoxy resin with the trade name ASSET 1011 provided by New Era Materials (Modlniczka, Poland) was applied as a polymer matrix. According to the MSDS from the resin producer, the resin mixture contained approximately 73 wt.% pure epoxy resin, <18 wt.% poly(ammonium phosphate), and 9 wt.% expanded graphite. The commercial polylactide in a powder form (PLA RXP 7501, MFI = 75 g/10 min, 190° C/2.16 kg) delivered by Resinex (Warsaw, Poland) was used as an additive of plant origin. Moreover, a twill weave fiberglass fabric that was 1000 mm wide and 350 g/m^2^ grammage, obtained from Rymatex company (Rymanów, Poland), was used as reinforcement. Fiberglass fabrics are made by interlacing the warp and weft in a chosen weaving style. The weave produced during weaving stabilizes the fibers in the material. 

### 2.2. Manufacturing of Composites

**Figure 1:**
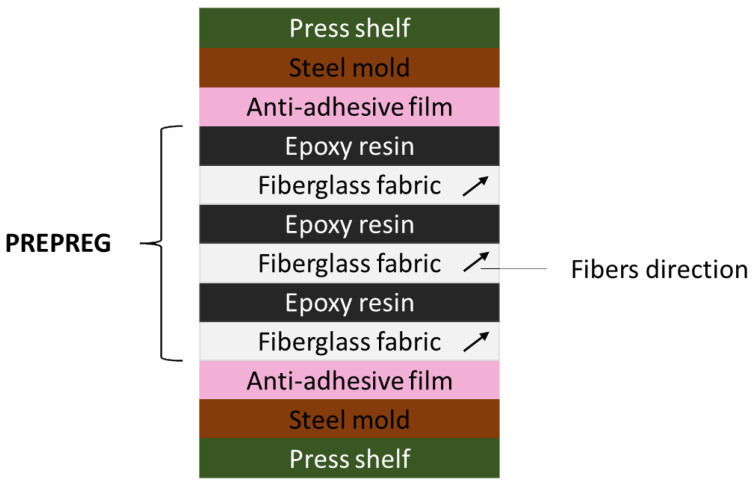
Method of preparing prepregs based on epoxy resin, which were cross-linked at 130 °C.

Before preparing the composites, the polylactide powder was dried in a laboratory dryer at 50 °C for 12 h. Then, after weighing the components for each composite, they were homogenized in a mortar for about 2 min. The next stage was to prepare the prepreg by stacking appropriate layers one after another, starting with a layer of fiberglass fabric, on which the first layer of resin with additives was poured through a sieve. It should be emphasized that each prepreg consisted of 3 layers of sieved resin and 3 layers of fiberglass fabric (maintaining the same direction of the fibers). This prepared composite was covered with two silicone (anti-adhesive) films and placed between two steel molds in a hydraulic press (Figure 1). The cross-linking process was carried out at a temperature of 130 °C and a pressure of 5 MPa for 8 min. Composites with dimensions of 8 × 8 cm were obtained. The weight composition of the produced materials based on epoxy resin is presented in Table 1.

The method of preparing these composites for testing the mechanical properties consisted of three stages. The first step was plasticizing the prepreg in a hydraulic press (90 °C, 6 bar, 5 min). Then, the prepreg was cross-linked in a second hydraulic press (130 °C, 10 bar, 8 min). The last stage was the curing of the composites in a laboratory dryer (136 °C, 45 min). 

### 2.3. Accelerated Aging Tests

Samples were subjected to solar aging in an Atlas SC 340 MHG solar simulator climate chamber (AMETEK Inc., Berwyn, IL, USA) equipped with a 2500 W MHG lamp. A unique range of solar radiation (UV, Vis, IR) was provided by a rare-earth halogen lamp. The prepared composites were exposed to a temperature of 70 °C with maximum solar radiation for the entire aging process of 800 h.

### 2.4. Contact Angle Determination and Surface Energy Calculation

A Dataphysics OCA 15EC goniometer equipped with a camera and SCA20 software (version 1.0) was used to determine the contact angles with distilled water, diiodomethane, and ethylene glycol. A minimum of six drops of each measuring liquid with a volume of 1 µL were placed on the surface of the tested materials from the side where the last layer was resin. Then, based on the contact angle values, the surface energy and its dispersive and polar components were calculated by applying the Owens–Wendt–Rabel–Kaelble (OWRK) method:

(1) $$E=E_{P}+E_{D}$$

(2) $$\frac{\sigma_{L}\left(1+cos\Theta \right)}{2\sqrt{\sigma_{L}^{D}}}=\sqrt{\sigma_{S}^{P}}\cdot \sqrt{\frac{\sigma_{L}^{P}}{\sigma_{L}^{D}}}+\sqrt{\sigma_{S}^{D}}\mathrm{as}\;a\;\mathrm{linear}\;\mathrm{function}\;Y\;=\;\mathrm{aX}\;+\;b$$

(3) $$Y=\frac{\gamma_{L}\left(1+cos\Theta \right)}{2\sqrt{\sigma_{L}^{D}}},X=\sqrt{\frac{\sigma_{L}^{P}}{\sigma_{L}^{D}}},a=\sqrt{\sigma_{S}^{P}},b=\sqrt{\sigma_{S}^{D}}$$

(4) $$E_{P}=a^{2}=\sigma_{S}^{P}\;oraz\;E_{D}=b^{2}=\sigma_{S}^{D}$$

### 2.5. FTIR Spectroscopy

Fourier-transform infrared spectroscopy was carried out using a Nicolet 6700 FT-IR spectrometer from Thermo Scientific (Waltham, MA, USA) equipped with a diamond Smart Orbit ATR sampling tool. The FTIR spectra were recorded in the range of 4000–400 cm^−1^ using 64 scans for all samples before and after 800 h of solar aging. Based on these spectra, the carbonyl index was calculated using the formula below:

(5) $$CI=\frac{I_{C=O}}{I_{C-H}}$$

### 2.6. Color Change Analysis

Color change measurements were carried out according to the PN-EN ISO 105-J01 standard [28] by using the UV-VIS CM-3600d spectrometer from Konica Minolta Sensing, Inc. (Osaka, Japan). Each sample was examined in at least three different places on the surface side with epoxy resin as the last layer. Then, based on the CIE-Lab system, the color of the samples was described by the three specific coordinates. The first was L, the brightness index, with a value range from 0 to 100, where 0 means black, and 100 is white. The second coordinate was the a* parameter, whose positive value means red, and the negative value means green. For the third parameter (b*), a positive value means yellow, and a negative one means blue. The color difference of the tested materials before and after solar aging was calculated based on the formula below:

(6) $$\Delta E=\sqrt{{\left(∆a\right)}^{2}+{\left(∆b\right)}^{2}+{\left(∆L\right)}^{2}}$$

### 2.7. Hardness Tests

Hardness tests were performed according to the PN-EN ISO 868standard [29] for all composites before and after 800 h of solar aging. Two digital hardness meters were applied, one on the Shore “C” scale and one on the Shore “D” scale (Zwick GmbH&Co, Ulm, Germany, pressure 50 N). 

### 2.8. Tensile One-Directional Test

**Figure 2:**
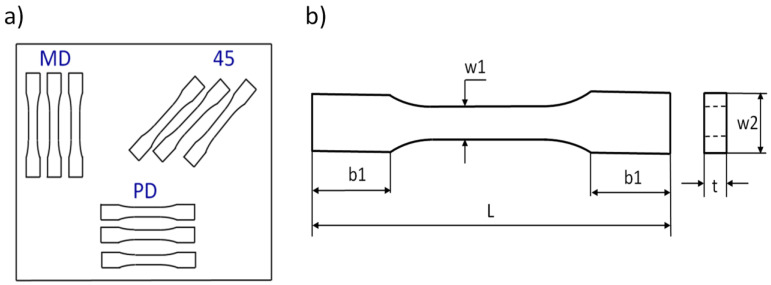
Set of cut samples (**a**) and sketch of sample subjected to tensile test (**b**).

The tensile tests were performed on an Instron testing machine model 4485. The tests of tension were conducted on the basis of the PN-EN ISO 527-1:2020-01 standard [30]. In accordance with this standard, the constant speed of moveable traverse was assumed to be equal to 2 mm/min. On this basis, the charts of force vs. elongation were obtained. The samples were taken according to scheme shown in Figure 2a, where symbols denote MD—main direction, PD—perpendicular direction, and 45—the angle with regard to the plate edges. The cutting out of the samples from plates was carried out using a Waterjet cutting machine. The dimensions of the samples were based on a standard: L = 80 mm, b1 = 15 mm, w1 = 5 mm, w2 = 10 mm, and average thickness t = 3 mm (Figure 2b). The Young’s modulus of composite was determined using a mechanical extensometer with a gauge length of 25 mm.

## 3. Results and Discussion

### 3.1. One-Directional Tensile Test before Aging

**Figure 3:**
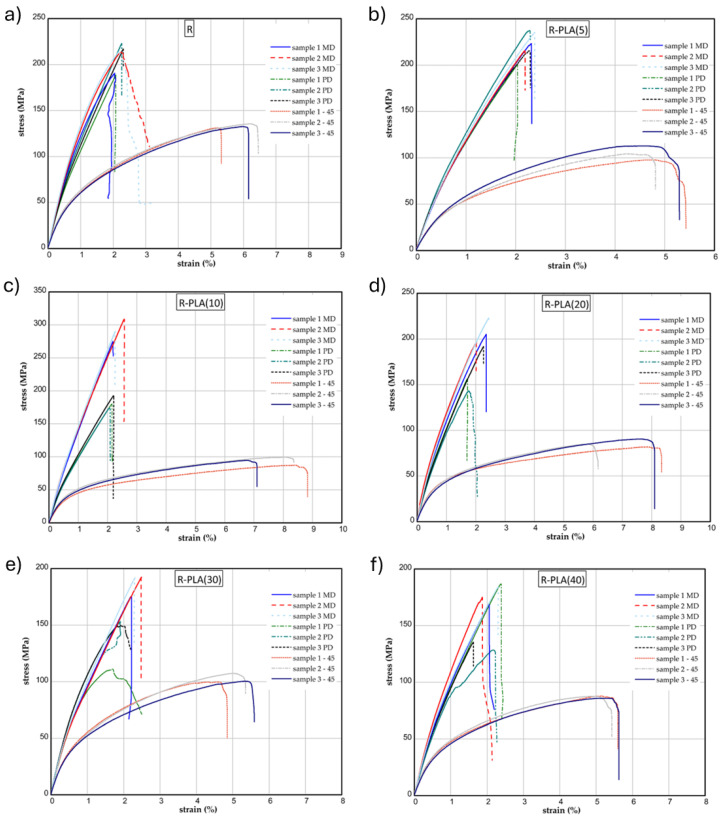
Curves of tension for reference material (R) (**a**), R-PLA(5) (**b**), R-PLA(10) (**c**), R-PLA(20) (**d**), R-PLA(30) (**e**) and R-PLA(40) (**f**).

The results of the tensile tests of some samples examined before the aging process are shown in Figure 3. The mechanical parameters of series were determined for three directions (MD, PD, 45). Figure 3a illustrates the strains expressed in % vs. normal stress in MPa for reference sample (R). The maximum stress and Young’s modulus for the MD samples were attained about 206 MPa and 16 GPa, respectively. In the case of the PD samples, the maximum stresses obtained were slightly higher or comparable (only 1–2% higher in comparison to the MD samples). In the case of the Young’s modulus, for MD samples, this parameter was higher. Related to the 45 samples, the mechanical parameters were lower (approximately 133 MPa and 9.7 GPa).

Analyzing the diagrams (Figure 3b–f), with the increase in the PLA presence in the samples, one can observe the visible rise in the strength and stiffness (R-PLA(5) and R-PLA(10)); but, the content of 20–40 wt.% of PLA significantly decreased the mechanical parameters (R-PLA(20), R-PLA(30), R-PLA(40)). Table 2 presents the determined values of the Young’s modulus and maximum stress for all the tested materials.

### 3.2. FTIR Spectroscopy

**Figure 4:**
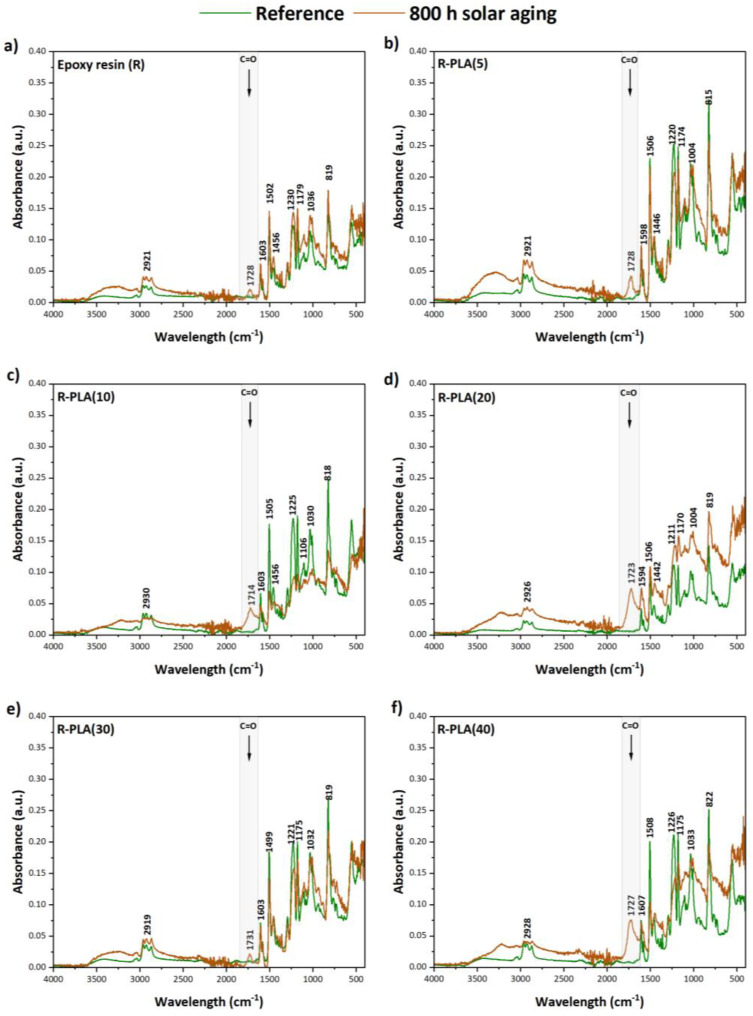
FTIR spectra of pure epoxy resin (**a**), epoxy resin-PLA(5) (**b**), epoxy resin-PLA(10) (**c**), epoxy resin-PLA(20) (**d**), epoxy resin-PLA(30) (**e**), and epoxy resin-PLA(40) (**f**), before and after 800 h of solar aging.

**Figure 5:**
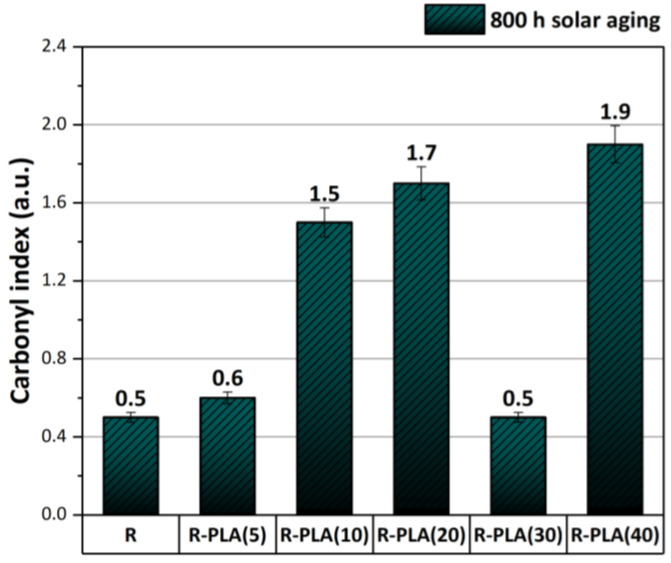
Calculated carbonyl index values obtained for composites based on epoxy resin after 800 h of solar aging. R—epoxy resin, PLA—polylactide.

The degree of the degradation progress of the tested composites based on epoxy resin filled with different amounts of polylactide (PLA) was assessed by the FTIR test. This was possible by analyzing the newly formed functional groups as a result of aging, the bands of which mainly indicated the presence of oxygen connections, such as C–O, C=O, O–C–O, and O–H. Figure 4 shows the obtained FTIR spectra of the tested materials. The pure epoxy resin (Figure 4a) was characterized by the presence of the following functional groups: O–H stretching and symmetric stretching of the primary amine (~3422 cm^−1^), C–H stretching of the epoxide group (~3037 cm^−1^) [31], C–H stretching of CH_2_ and CH_3_ aromatic and aliphatic (~2962–2869 cm^−1^) [32], C=C stretching of the aromatic ring, N–H bending of the primary amine (~1603 cm^−1^) [33], N–H bending of the primary amine (~1581 cm^−1^), C–C stretching of the aromatic ring (~1502 cm^−1^) [31], deformation C–H of CH_2_ and CH_3_ (~1456 cm^−1^) [34], C–O–C stretching of the ether linkage (~1230 cm^−1^) [31], C–O stretching of the aromatic ring (~1179 cm^−1^), aromatic stretching (~1105 cm^−1^) [33], C–O–C stretching of the ether linkage (~1036 cm^−1^) [35], C–O–C stretching of the oxirane groups (~825 cm^−1^) [36]. As was observed, the addition of PLA to the epoxy resin did not cause structural changes. The obtained FTIR spectra are characterized by the same functional groups; but, they differ in the case of some peaks only in the absorbance intensity. Nevertheless, after 800 h of solar aging, the greatest change was observed for the peak at around 1720 cm^−1^, which corresponds to the formation of carbonyl groups (C=O). Based on the differences in the intensity of the peaks at the wavelength of about 2920 cm^−1^ and 1720 cm^−1^, the carbonyl index values were calculated for all the tested composites, which show the progress in the degradation process of polymeric materials (Figure 5). The highest values of this parameter were recorded for the samples of epoxy resin containing polylactide in the amount of 20 and 40 phr, which means that they were the least resistant to the set conditions during solar aging. In addition, based on this study, it can be concluded that increasing the amount of bioadditive (polylactide) in the polymer matrix adversely affects the preservation of the properties of the epoxy resin and accelerates its degradation process, with the exception of the sample containing 30 phr of PLA, which could be caused by, among other things, the insufficiently distributed additive in the polymer matrix or its location in the solar chamber.

### 3.3. Contact Angle Determination and Surface Energy Calculation

The surface wettability test of composites before and after 800 h of solar aging was carried out using distilled water and diiodomethane as measuring liquids, and based on the determined contact angles, the surface energy and its components (dispersive and polar) were calculated. For each material, two paddle-shaped samples were examined by placing six drops of each measuring liquid on them in different places from the side where the last layer was resin.

**Figure 6:**
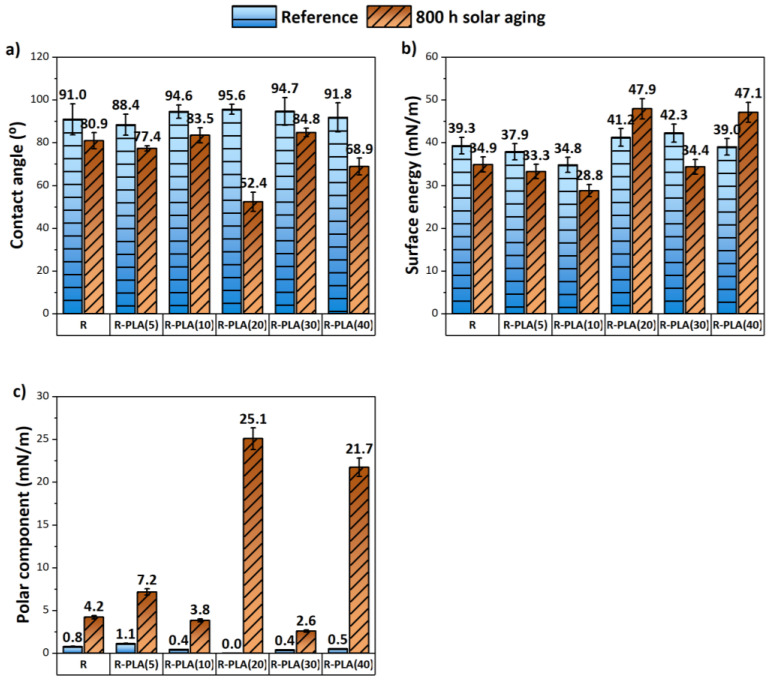
Average values of water contact angles before and after 800 h of solar aging (**a**) and the changes in surface energy (**b**) and its polar component (**c**) caused by the accelerated aging tests. R—epoxy resin, PLA—polylactide.

Figure 6a presents the obtained contact angle values in the case of water used as the measuring liquid. Before aging, most of the tested surfaces of the samples showed a hydrophobic character, because their contact angles were higher or close to 90°. On the other hand, after 800 h of solar aging, it was possible to observe that their surfaces became more hydrophilic, as evidenced by lower values of contact angles. The greatest change in surface wettability was noted for the sample containing 20 and 40 phr of polylactide. This testifies to their good wettability but also to the fact that they were the most susceptible to oxidation processes. For the same composites, the highest increase in surface energy and its polar component was also observed, which is shown in Figure 6b,c. For the sample of epoxy resin with the addition of 20 phr of PLA, the surface energy value after the aging process was 47.9 mN/m, and its polar component was 25.1 mN/m. During controlled aging processes, appropriate functional groups (e.g., hydroxyl) are formed on the surface of polymeric materials, which are closely related to their degradation processes. In general, it can be stated that the higher the amount of bioadditive used in the resin composite, the lower its resistance to aging processes; the exception was the material with the addition of polylactide in the amount of 30 phr. For this sample, smaller changes in the contact angle and the polar component of surface energy values were observed after 800 h of solar aging, which means that fewer hydroxyl groups were formed on its surface, and thus it was more resistant to oxidation processes.

### 3.4. Color Change Analysis

**Figure 7:**
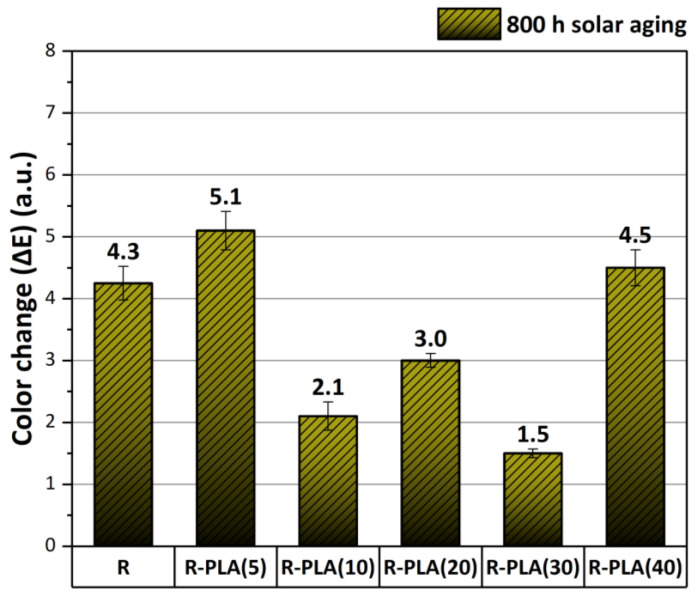
Color change (ΔE) in materials caused by 800 h of solar aging. R—epoxy resin, PLA—polylactide.

A color change in polymeric products can be the first visual indication of the degradation process. Therefore, the color change in the composites based on epoxy resin was tested after 800 h of solar aging. Based on the spectrophotometric measurements and the CIE-Lab space, three color coordinates were determined: L*—lightness index, a*—axis for which a positive value means red and a negative means green, and b*—axis for which a positive value means yellow and a negative means blue. Moreover, on the basis of these parameters, the color change (ΔE) was calculated. The obtained results are shown in Table 3 and Figure 7. 

For most samples, a decrease in the L* values was observed as a result of 800 h of solar aging, which means that they became darker. Additionally, when analyzing the values of the a* and b* parameters before and after aging, it can be noticed that the coloration of these materials shifted more toward red and yellow shades. The smallest color change was observed for the sample with the addition of polylactide in the amount of 30 phr (ΔE = 1.5), which also showed the highest resistance to the oxidation process based on the surface wettability test and the FTIR study. In accordance with the principle that a color change greater than five can be easily noticed visually by the observer, in this case, this difference was visible for the composite with the addition of 5 phr PLA (ΔE = 5.1).

### 3.5. Hardness Tests

**Figure 8:**
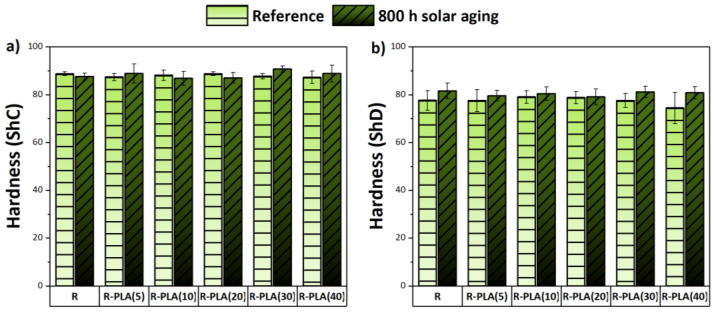
Hardness tests performed for pure epoxy resin and filled with polylactide on the Shore “C” scale (**a**) and on the Shore “D” scale (**b**) before and after solar aging. R—epoxy resin, PLA—polylactide.

The hardness of the epoxy resin composites was tested in six different places, both on the surface where the last layer was epoxy resin and on the side where the last layer was a fiberglass fabric. The obtained results of the hardness in the Shore scales “C” and “D” are shown in Figure 8a,b. As a result of solar aging, most of the samples slightly increased their hardness, which may indicate the cross-linking process during their stay in the chamber. Moreover, it can be seen that the addition of polylactide to the epoxy resin did not significantly affect the value of this parameter. It should be emphasized that all the composites showed higher surface hardness from the side where the last layer was fiberglass fabric, their Shore “C” hardness was about 88 ShC, and their Shore “D” was about 77 ShD. This means that these materials were very hard because when the Shore “D” hardness is greater than 60 ShD, the sample is stiff, which would be ideal for applications, where such durability is required.

## 4. Conclusions

The aim of this work was to obtain epoxy-based composite structures with good mechanical performance, high aging resistance, and an improved degradability profile. Therefore, powdered polylactide in the amount of 5, 10, 20, 30, and 40 phr was introduced into the epoxy resin, and the composites were fabricated by a simple method, which is similar to that used on an industrial scale in the fabrication of these products.

Based on the one-directional tensile test performed before the aging processes, it was observed that the addition of polylactide in the amount of 5 or 10 phr caused a significant increase in strength and stiffness, while its higher content (20, 30, or 40 phr) led to a deterioration in the mechanical parameters.

The next stage of the research was the analysis of the effect of the applied biofiller on the aging resistance of the epoxy resin. Samples were aged in a solar chamber for 800 h at a temperature of 70 °C. It was noticed that composites containing 20 or 40 phr of PLA were characterized by the highest susceptibility to the solar aging process. Therefore, considering all the research carried out, it was concluded that the optimal amount of PLA filler in the epoxy resin composite should not be greater than 10 phr to maintain its mechanical behavior and high aging resistance.

To the best of our knowledge, the influence of polylactide as a biofiller on the general characteristics of epoxy resin has not yet been described in the literature. Therefore, this work perfectly fills existing gaps and may contribute to the wider use of additives of natural origin, which may constitute an excellent alternative to commonly used non-renewable compounds.

## Figures and Tables

**Table 1:** Weight composition of the materials filled with polylactide.

Mixture	Weight Composition [phr]	
	Epoxy Resin (ASSET 1011)	Polylactide (PLA RXP 7501)
1	100	-
2	100	5
3	100	10
4	100	20
5	100	30
6	100	40

**Table 2:** Main values of Young’ modulus and maximum stress.

Sample	Young’s Modulus [GPa]			Maximum Stress [MPa]		
	MD	PD	45	MD	PD	45
R	16.11 ± 0.40	14.38 ± 0.74	9.74 ± 0.38	205.8 ± 13.2	209.6 ± 18.4	133.1 ± 2.2
R-PLA(5)	15.64 ± 0.45	14.98 ± 0.15	8.77 ± 0.31	218.0 ± 18.2	224.6 ± 9.9	105.0 ± 7.6
R-PLA(10)	17.25 ± 0.51	13.63 ± 0.59	8.64 ± 0.40	291.3 ± 16.9	182.2 ± 9.4	93.9 ± 6.2
R-PLA(20)	15.25 ± 1.63	12.82 ± 0.92	7.58 ± 0.50	208.3 ± 13.3	163.9 ± 25.1	85.7 ± 4.4
R-PLA(30)	13.35 ± 0.30	13.82 ± 0.64	8.93 ± 0.11	186.6 ± 9.5	138.0 ± 23.4	102.4 ± 4.2
R-PLA(40)	12.96 ± 0.65	12.20 ± 0.54	7.76 ± 0.23	176.0 ± 7.7	150.1 ± 32.0	87.1 ± 1.1

**Table 3:** Color parameters (L*—brightness index, a*—red–green axis, b*—yellow–blue axis) determined in the CIE-Lab system before and after solar aging, on the basis of which the color change (ΔE) was determined.

Sample	Before Aging			After Solar Aging		
	L*	a*	b*	L*	a*	b*
R	37.3	−0.2	1.4	33.5	0.2	2.6
R-PLA(5)	38.9	−0.3	1.5	37.2	0.5	4.9
R-PLA(10)	35.4	−0.1	0.9	34.9	0.0	2.6
R-PLA(20)	36.2	−0.4	1.1	36.6	0.4	3.8
R-PLA(30)	37.3	−0.3	1.4	37.0	0.1	2.4
R-PLA(40)	38.5	−0.5	1.4	39.1	0.4	4.0

## Data Availability

Data are contained within the article.
